# How Does Supermarket Category Management Shape What Is on Supermarket Shelves and Influence Diet and Health? Secondary Analysis of Qualitative Interviews With Retailers and Suppliers

**DOI:** 10.34172/ijhpm.8932

**Published:** 2026-03-10

**Authors:** Roxanne Armstrong-Moore, Michael Benson, Martin White

**Affiliations:** ^1^MRC Epidemiology Unit, School of Clinical Medicine, University of Cambridge, Cambridge, UK.; ^2^Sheffield Business School, Sheffield Hallam University, Sheffield, UK.

**Keywords:** Supermarkets, Food Marketing, Category Management, Food Environment, Unhealthy Diets, United Kingdom

## Abstract

**Background:**

Unhealthy diets drive high rates of non-communicable diseases. Many food environments are dominated by foods high in fat, salt, and sugar (HFSS). Supermarkets are a predominant source of groceries, and the relationships between retailers and their suppliers determine the foods displayed on their shelves. The disproportionate display of less healthy foods suggests that existing regulatory frameworks are sub-optimal for public health. We aimed to investigate the nature of relationships between supermarkets and their suppliers in the UK, and their implications for dietary public health.

**Methods:**

We undertook secondary analysis of in-depth interviews with UK retailers and suppliers (n = 19), using inductive and deductive approaches to thematic analysis, underpinned by our quest to understand how food retailing drives less healthy diets. Codes and themes were generated and refined, then mapped diagrammatically and are presented in an explanatory narrative.

**Results:**

Large suppliers are critical to the supplier-retailer relationship, dominating category management and supermarket shelves. The relationship brings mutual benefits for supplier and retailer. Category managers from large suppliers engender indebtedness among retailers by building rapport and trust, and investing financially and materially in retailers. Reciprocity by retailers is enacted with preferential contracts and the award of leading roles in category management ("category captaincy"). Large suppliers thus gain competitive advantage, with preferential access to premium shelf space, driving greater sales. Important positive reinforcing feedback loops maintain the relationship, described as a "virtuous circle." Yet, there are also countering forces, which act as negative feedback loops.

**Conclusion:**

Where the retailer-supplier relationship involves the largest manufacturers, it drives the product mix and volumes on supermarket shelves, resulting in a disproportionate dominance of the largest, processed food brands. The nature of this relationship is likely a key factor preventing movement towards an overall healthier food offer to consumers and remains a public health concern.

## Background

**Key Messages**

**Implications for policy makers**

- Over 80% of UK groceries are bought from supermarkets. There is overall a disproportionate prominence of less healthy foods on supermarket shelves, in terms of volume and mix visible to consumers. Newly introduced regulations on the placement and promotion of foods high in fat, salt, and sugar (HFSS) in UK supermarkets are changing the arrangement of foods, but observations suggest adaptations are taking place, resulting in novel and prominent displays of less healthy foods that are not covered by the regulations.
- Supermarkets control what consumers sees on shelves, and our findings suggest this is importantly influenced by the relationships between supermarkets and the largest manufacturers. This study provides evidence that the Groceries Supply Code of Practice (GSCOP), which governs relationships between suppliers and retailers, and aims to ensure fair competition, does not serve the interests of public health. Cross-government review of this regulation as part of Department for Environment, Food & Rural Affairs’ (Defra’s) national food strategy is needed to improve access to healthier foods.
- The prominence of unhealthy foods in grocery retailing is not unique to the UK, so our findings have implications for policy internationally.

**Implications for the public**

Supermarkets decide what foods go on their shelves and in which proportions. However, supermarkets work disproportionately with representatives from the largest manufacturers to decide how foods are displayed in each food category, in order to maximise sales. This strategy is described by the industry as a “triple win” – for the manufacturer, supermarket and customer. Supermarkets are laid out in ways that manipulate consumers and promote unhealthy food choices. Strategic placement of items, including large and prominent displays and products at eye-level, lead to greater sales. Customers have expressed concern with such marketing strategies, which lead to unhealthy food purchases that adversely affect diets and health. The relationship between manufacturers and supermarkets is regulated in the UK to ensure competition is fair. We analysed interview data from industry experts in suppliers and retailers, which revealed problematic aspects of this relationship that are likely preventing movement towards a healthier food offer, and are therefore a public health concern.

In the United Kingdom, in common with most high-income countries, people are exposed to food environments that promote excessive intakes of unhealthy processed foods.^1^ Humans have evolved taste preferences for sugary, salty and fatty foods^2^ and industry has recognised the potential to exploit this, in particular through the development and marketing of highly palatable foods that are high in salt, unhealthy fats, sugar, and calories. The commercial food system plays a key role in determining such food environments.^3^ A dominant commercial emphasis on added-value processed foods is reflected in the range and prominence of foods in supermarkets. Sales of less healthy foods are increasing globally, and are associated with rising levels of overweight,^4^ as well as mortality from cardiovascular disease, diabetes and cancers.^5,6^

 The processed food manufacturing sector (hereafter referred to as “suppliers”) is largely oligopolistic, dominated by a small number of large manufacturers, and presents significant barriers to new entrants.^7^ Whilst supermarkets have made some attempts to promote healthier shopping habits, they are often vague and inconsistent, and their systemic impacts are unknown.^8^ Supermarkets have also been shown to use marketing tactics to encourage shoppers to purchase unnecessary items, with unhealthy foods tending to dominate shelf space and having a higher chance of being offered at a discount.^9^

 The relationship between suppliers and retailers, in particular supermarkets (the source of 82% of food purchases in the UK^10^), determines what is on supermarkets shelves. In May 2006 the UK Office of Fair Trading (a non-ministerial government department, in existence from 1973-2014) asked the Competition Commission (now the Competition and Markets Authority [CMA]) to investigate and report on the supply of groceries by UK retailers. Reporting in 2008, the Competition Commission concluded that the working methods of supermarkets adversely affected competition. Following this, the Groceries Supply Code of Practice (GSCOP)^11^ was enforced, providing a regulatory code for supermarkets with the intention of ensuring fairer relationships between retailers and suppliers. The GSCOP regulates retailers trading in the UK with an annual grocery turnover of more than £1 billion and, at the time of writing, covers the following designated retailers: Aldi Stores Ltd; Amazon.com, Inc; Asda Stores Ltd; B&M European Value Retail SA; Co-operative Group Ltd; Iceland Foods Ltd; J Sainsbury plc; Lidl GB Ltd; Marks and Spencer plc; Ocado Retail Ltd; Tesco plc; TJ Morris Ltd (trading as Home Bargains); Waitrose Ltd and Wm Morrison Supermarkets Ltd. The GSCOP states that retailers: (*a*) must not directly or indirectly demand payments from suppliers towards marketing costs; (*b*) must not directly or indirectly require suppliers to make payments in order to secure better positioning of their products within a store; and (*c*) must not require suppliers to fund the cost of promotions of their products in store.

 In the US, it has been reported that the relationship between suppliers and supermarkets is anti-competitive and this is a key reason why supermarkets are dominated by unhealthy food products.^12^ Rivlin describes the use of “category captains” (a representative of the supplier who works closely with retailer category managers within supermarkets) as to ensure preferential access to shelf space, in order to maximise sales and profits. Category captains work with the retailer to perform research and planning of a category (eg, chocolate confectionery) and this means arranging the location of not only their own products, but also those of competitors. Alan et al studied the impact of category captains on sales in the US and found that their interventions improve category sales, and that, while some competing suppliers do also benefit from the category captain, others suffer.^13^ It is mainly larger, dominant brands with either the most, or second most sales who are selected by the retailer to take on the role of category captain.^14^ In the US, it is accepted that category captains pay retailers to be in the position and therefore there must be a benefit to the role in terms of preferential treatment by the retailer and benefits to their brand at the expense of competitors.^15^

 Benson has reported a contemporary understanding of the category management and captaincy arrangements in the United Kingdom after the introduction of the GSCOP, suggesting it aids more collaborative interactions between suppliers and retailers.^16^ Category managers are appointed by both retailers and suppliers and work collaboratively to maximise sales within their category within a particular retailer, particular stores and, in the case of suppliers, across multiple retailers. Category managers from suppliers taking on a dominant category management role within a particular retailer may be designated the role of category captain, sometimes known as preferred supplier or category leader.

 Despite detailed understanding of the category management relationships in the UK from a business perspective, the implications of the current arrangements for food access, diet and health are unclear. Less healthy foods are more often promoted within supermarkets,^17^ and reducing the placement of unhealthy foods on aisle (gondola) ends and at checkouts results in healthier food purchases.^18^ As a result, the placement of foods high in fat, salt, and sugar (HFSS) in high footfall areas, such as front of store, ends of aisles and checkouts was banned in all supermarkets in England from October 2025.^19^

 Nevertheless, this only deals with one aspect of product placement. The number of facings (appearances) of a product on shelves within aisles boosts sales, as does placement of products at eye level.^20^ Prominent placement and increased availability of less healthy products drives purchases and can therefore lead to less healthy diets.

 In this study, we undertook secondary analysis of existing data from interviews with suppliers and retailers concerning the category management relationship^16^ to investigate the following questions:

1. What is the nature of the relationship between suppliers (producers and manufacturers), and grocery retailers (ie, contractually, and in terms of implicit and explicit expectations)?
2. What are the implications of this relationship for competition, and for the healthiness of the overall food offer in grocery retailing?
3. What are the implications of the GSCOP and The Groceries (Supply Chain Practices) Market Investigation Order 2009 for this relationship and for public health?

## Methods

###  Design

 We undertook a qualitative study, in which we conducted secondary analysis of semi-structured interviews with suppliers, retailers and industry associates undertaken in 2016-2018. We used a combination of inductive and deductive thematic analysis, together with diagramming. The data was viewed through a public health lens, consistent with our aim to explore the implications of business practices for public health.

###  Interviews

 Participants were interviewed by MB, to inform a narrative on value creation in category management in UK supermarkets.^16^ Interviews were carried out between November 2016 and February 2018. The sample comprised 19 interviews: 12 staff from suppliers, and 7 from retailers, or retailer industry bodies. Participants were from small (<£100 million sales value per annum), medium (£100-250 million) and large (>£250 million) companies. Interviews included grocery retailers that are subject to the GSCOP. We deidentified interviews to maintain anonymity, indicating alongside quotes only whether the interviewee was from a large, medium or small supplier or retailer.

###  Analysis

 Two co-authors undertook the secondary analysis of interview data (RM and MW). In the first phase, we undertook inductive analysis following the phases outlined by Braun and Clarke^21^: (*i*) familiarisation of transcripts; (*ii*) coding; (*iii*) generation of initial themes; (*iv*) developing and reviewing themes; (*v*) refinement, definition and naming of themes; and (*vi*) write up of themes. Codes and themes were generated without the application of an *a priori* coding framework and not informed by previous research or existing theory but guided broadly by our three research questions. This allowed us to apply an exploratory lens to data that was collected for a different purpose. However, the researchers went into the analysis of this data with some prior knowledge of the subject, both from a commercial and public health perspective.

 In the second phase, we then used diagramming^22^ to develop a conceptual understanding of the process of category management, as well as its underlying drivers and motivations, constraints and consequences. A conceptual map of the category management system was developed using Kumu software.^23^ The detailed content of the themes identified from the deductive analysis, together with supporting quotations enabled us to derive “nodes” – defined elements of explanatory pathways – and the direction (uni- or bi-directional) and polarity (positive/enhancing; or negative/dampening) – of relationships between nodes.

 In the final phase, we applied a deductive approach to the data, exploring the inductively generated themes and the diagram to answer the three research questions. Further interpretation of the findings is addressed in the Discussion.

###  Positionality

 Both (RM and MW) are public health researchers with an interest in diet, food systems and public health and the commercial determinants of health,^24^ and therefore have prior experience and knowledge within the field. They led the qualitative secondary analysis. MB subsequently contributed to interpretation, and is a researcher with a primary interest in marketing and business studies, who conducted the interviews.

## Results

 Six interviewees were from small companies, two from medium sized companies, and eleven from large companies. Four themes were found: (1) Characteristics of a successful category management relationship between retailer and supplier; (2) The virtuous circle of Category Management; (3) The constraining influences of regulation and commercial risk on category management; and (4) Competitive advantages of category captaincy and consequences for food purchasing, diet and health. The conceptual diagram of the category management system, based on this inductive analysis, is shown in the Figure, and will be referred to in the narrative concerning the themes below to avoid repetition.

**Figure:**
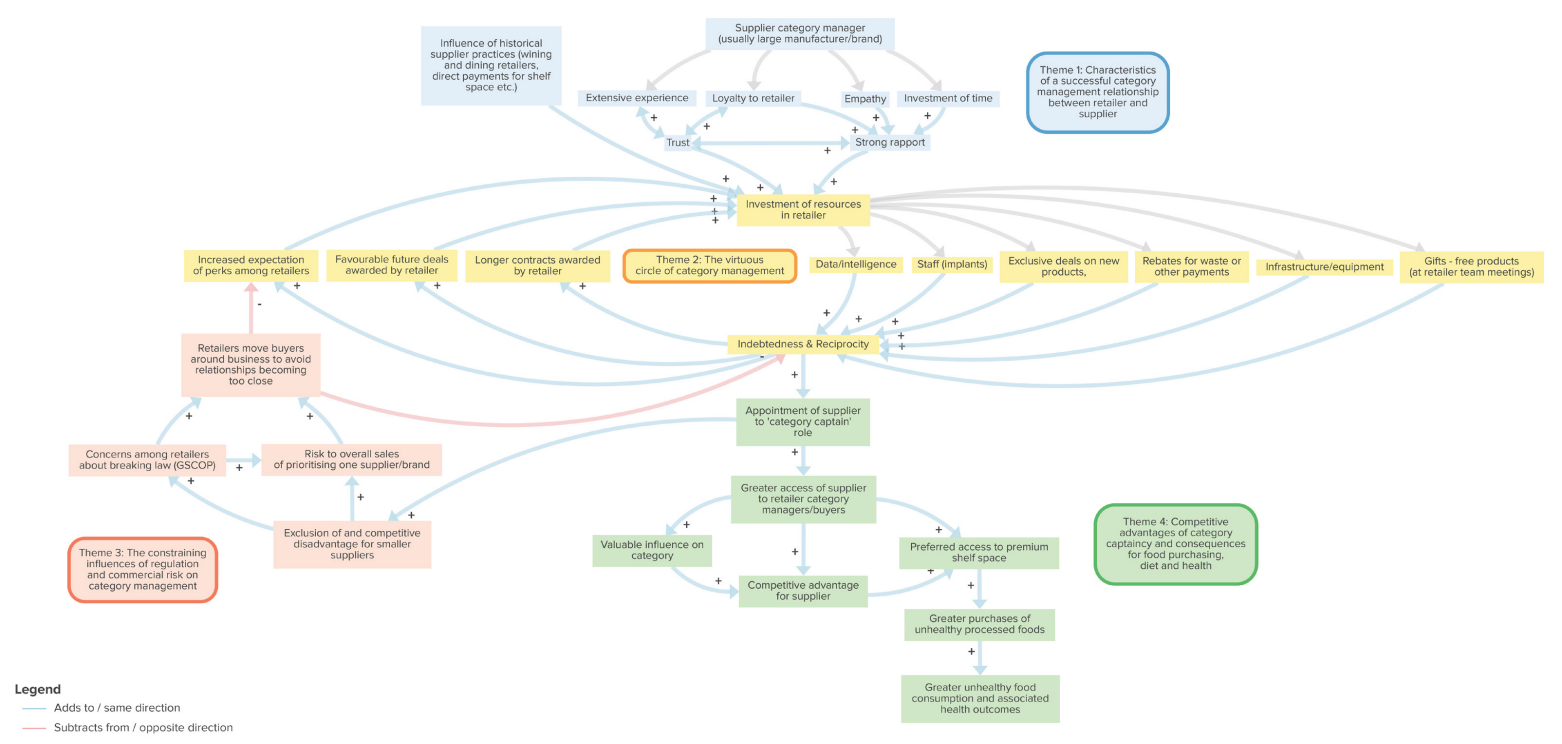
Conceptual Map of the Category Management System in UK Supermarkets, Derived From Thematic Analysis of Interviews With Suppliers and Retailers.

###  Theme 1: Characteristics of a Successful Category Management Relationship Between Retailer and Supplier

 The nodes relating to theme 1 are shown in blue in the Figure. Participants emphasised important aspects of a successful relationship between suppliers and retailers. We found evidence that relationships were complex and transactional, with an emphasis on relationships gelling, empathy and investing emotionally in the relationship:

 “*As a category manager I think that as you spend so much time with the merchandisers they have got to like you, […] I think personality, the way you gel with them is important to build up trust”* (Supplier 1, medium company).

 “*For me and anyone in our team, understanding who the other person is across the table and having empathy for them is really important. So that is what a lot of our emotional intelligence training is designed for, talking about adult-to-adult conversations because you recognise you are both people across the table”* (Supplier 9, small company).

 Relationships were importantly influenced by the amount of experience of the supplier and the degree of loyalty to the other party, both of which built trust, which in turn led to greater loyalty and enabled building of experience (shown by bi-directional relationships in the Figure):

 “*Once a supplier has demonstrated they are trustworthy it does lead to loyalty. As buyers move around organisations, they will keep portfolios of companies they have worked with successfully and reward the companies again later with more business; hence the buyers remain loyal to them”* (Retailer 5, large company).

 One supplier explained that the amount of time spent with a merchandiser emphasised the importance of being liked, and this led to trust. The same supplier also explained that it is important to be doing business with people you like spending time with, and that offering support without the expectation of a return helps to build strong rapport:

 “*I think relationships in general, and this is something that I have been taught, is ‘people buy people,’ so you like doing business with people you enjoy being with, and I think it’s about offering without expecting a return; and this is a good way to build a relationship. So, by being in a relationship try and help the customer get a ‘win’ rather than doing it just for yourself. So put chips in the ‘emotional bank account’ and so invest in the relationships without expecting anything back”* (Supplier 1, medium sized company).

 Believability and “speaking the same language” were also highlighted as important personality traits for supplier category managers:

 “*If you are the most inspiring and you are the most believable and you are the one who is really talking the retailer’s language the best, you are more likely to get your recommendations away than your competitors”* (Retailer 5, large company).

 Interestingly, the closeness of rapport at the individual level was sometimes more important than the commercial relationship:

 “*On the other side we have clients such as [supermarket name]. We don’t have a good relationship with them, for 3 years we have not had a supplier plan, but they call me in personally and want me to advise them on the category. This will be before any commercial proposals go to them from us. This is because this relationship is more personally driven, rather than being [supplier] driven” *(Supplier 6, large company).

 Lastly, there were comparisons between the *“good old days” *and now, with an indication that something has changed, although it was not entirely clear what. The suggestion seemed to be that, although the closeness of retailer-supplier relationships is deemed vital, these are not as close as they used to be. This historical context of category management appeared in the discourse of a number of interviewees, suggesting that historical practices remain an important influence on current behaviour:

 “*Sometimes you actually work inside the retailer, so if you are working on projects, you may be asked to sit there. In the good old days [we] used to sit in [supermarket name]. So actually, being in the office, go for lunch with them and become a part of the team forges you much closer together … I think there is less of that now, but there is certainly something to be said about being there, a friend and being available”* (Supplier 8, large company).

 Regardless of this historical context, it was suggested that the culture of category management in smaller companies is significantly different from larger companies, with a greater focus on authenticity and passion for their products:

 “*Before I joined the industry, I am quite old-fashioned, but I had ingrained in me what old school selling looked like, taking them out to nice restaurants for wining and dining. I am sure that side of things is less prominent for us than the bigger companies. For us, buyers are busy people and their time is precious, so we don’t want to spend our time not talking about the exciting things, we are not the norm but are foodies wanting to talk about our passion. We don’t wear suits, don’t roll up with decks of slides, we will do food safaris with them rather than wining and dining; we will engage their teams and do fun things themed around their category”* (Supplier 9, small company).

###  Theme 2: The Virtuous Circle of Category Management

 Nodes in theme 2 are coloured yellow in the Figure. The development of a successful category management relationship between a supplier and a retailer was further cemented by the investment of resources in category management within the retail environment by the supplier. Six key types of investment were identified in interviews with both suppliers and retailers: (1) the provision of market intelligence in the form of sharing of data with the retailer by the supplier; (2) seconding staff to retailers, sometimes called “implants”; (3) offering exclusive deals on new products to a specific retailer; (4) offering financial support, such as rebates for volume sales, compensation for wastage etc; (5) the provision or funding for in-store infrastructure or equipment (eg, marketing displays); (6) offering gifts to retail staff, for example free product at team meetings.

 Data was seen as important, but only in the context of extensive experience in a supplier’s category manager:

 “*Recommendations will need, where possible, to be backed up by data, but within the trusting relationship there will also always be a ‘leap of faith’ if the manufacturers are doing their job correctly. As long as you can back it up with a number of things then it is fine. […] The trick is to put context behind why this is a good idea and applying it to the category, for example based on your perception of the market which if backed by data cannot be wrong, it is your take. Experience is also important and having been ‘around the block’”* (Retailer 6, small company).

 Some supermarkets were reported to have many staff seconded by suppliers to help advise on category management. However, not all suppliers are able to afford such resources, and supermarkets will draw on advice from multiple suppliers, even within a category:

 “*If you think about say [supermarket name] as a good example the amount of implants they have got. These are say [brand] employees who sit in [supermarket name] and help the buyer to manage the category. This provides them with head count they don’t have to pay for, only one category captain can access their data on [supermarket specific data portal]”* (Supplier 1, medium company).

 “*No. We don’t use implants. You need to be a much bigger organisation to do that, you know, I believe companies that do that can gain an advantage” *(Supplier 2, small company).

 “*That however does not prevent other suppliers from contributing category advice. [supermarket name] will definitely ask other suppliers to contribute even though we have several implants working within their organisation”* (Supplier 1, medium company).

 An exclusive deal was framed by one large supplier as about joint value creation:

 “*I will give you an example of what we would call joint value creation where we work with the retailer to bring new products to market. We have recently worked with [supermarket name] to bring a product to market that absolutely meets the target shoppers. … we have worked with them to plan that launch and actually created something exclusive … they actually get it for a 6-month exclusivity deal to offer a point of differentiation to their shopper and we would support them by bringing shoppers into store by investing in their media … That is a good example of how we have worked together to plan that launch against a joint shopper need”* (Supplier 8, large company).

 Food manufacturers are expert at marketing and these techniques primarily designed for consumers were also used with retail staff:

 “*Or we will do a head office event where we will do similar; next week for example we have an event at [supermarket name] head office where we will all be there including [the managing director]; anyone of my level will be there in branded t-shirts giving out free product to the [supermarket name] staff”* (supplier 6, large company).

 The provision of such resources to retailers by suppliers generates indebtedness and leads to reciprocity in a number of forms, which may include longer contracts awarded by retailers and more favourable future business deals.

 “*Once a supplier has demonstrated they are trustworthy it does lead to loyalty. As buyers move around organisations, they will keep portfolios of companies they have worked with successfully and reward the companies again later with more business; hence the buyers remain loyal to them”* (Retailer 5, large company).

 It can also lead to a greater expectation of “perks” (investment of resources) from suppliers.

 “*But it has almost got to a point where I think it is seen as a bit of a perk that ok Mr. [supplier brand] that I do £60m of business a year with you, or more even … and as part of that business relationship I want you to pay for someone to sit in my office which I can use to do with them whatever I want…”* (Supplier 3, small company).

 The mutual benefits of the relationship were described as a virtuous circle, and this retailer talked of retailers and supplier being locked into the relationship with “golden handcuffs”:

 “*We would reward in a number of different ways. So they would be offered any new opportunities, offered the longest term contracts, so if a buyer thinks there is value in really locking down here. That only happens once the trust is built, and a trusting relationship exists. So as a supplier where trust is high, the buyer rewards with loyalty, and you can secure greater value for your business. So, this gives the confidence for the supplier to invest in their business ie, a new piece of equipment and rest in the security a long-term contract exists with the retailer. Then, of course it becomes quite a virtuous circle, so as a buyer you have jointly created this good relationship, there has been an investment in infrastructure, and you suddenly realise without that infrastructure there that the supply chain will not work as well. Therefore, that kind of locks the 2 parties that unless somebody else is going to come along with a boat load of money to invest the same then we are reliant on the trusted partner. So, it can become a situation of ‘golden handcuffs’”* (Retailer 3, large company).

 This highly transactional relationship between retailer and supplier means both parties hope to create value for the other, and ultimately for the consumer. The relationship was described as a “triple win”:

 “*I would define CM [category manager] as a […] marketing process, it is a process for the three parties to get together to drive category value growth. We talk about a theory called the ‘triple win’ […] So, the industry of CM grew up around this notion. Wouldn’t it be great if we knew what consumers might need, and also wouldn’t it be great if we understood the shopper as they might be quite different to the consumer? So, the consumer, the people that eat it, different from the buyers so we need to understand better how people shop in the supermarket; nowadays [also] online to understand how to get them to buy more”* (Supplier 8, large company).

###  Theme 3: The Constraining Influences of Regulation and Commercial Risk on Category Management

 Nodes in theme 3 are coloured orange in the Figure. While the virtuous circle of category management is characterised by positive or reinforcing feedback loops, there is also a set of factors that represent negative or balancing feedback loops. These are constraining influences that diminish the power of reciprocity.

 There is a risk to a retailer if they prioritise the advice of one supplier, as broader market intelligence may be of greater benefit to overall sales.

 “*Traditionally it’s been more the largest supplier within the category who tends to have that collaborative relationship with the retailer, but I think retailers also now feel that is dangerous to take just one view, and as objective as it may appear it is better to talk to other suppliers within the category to validate the first recommendation and even come up with some additional and innovative ideas to supplement it”* (Retailer 6, small company).

 For this reason, supplier category managers are expected by retailers to give advice on a category as a whole, and this can sometimes lead to suppliers making sacrifices concerning their own brand in order to drive the entire category:

 “*Probably the most important thing is to be category focused. Not brand focused […] sometimes we have to recommend things for the category which are not right for our business […] you would possibly focus on other brands before your own. We would talk about other brands and point out if they had a gap in their portfolio, if they were struggling in a particular segment and there was a gap and could be filled by a competitor, we would recommend it as we are driving category and not specifically our own brand”* (Supplier 4, large company).

 Impartiality was described as important in this regard. Suppliers are expected to remain objective, and to do the best thing for the category overall. Retailers described the importance of trust, with suggestions that with free reign and no trust, some suppliers would only put their products on the shelves:

 “*That is interesting as to build the trust more do you actually become a bit biased the other way, ie, biased towards the other suppliers’ products. So, you appear more impartial even though it’s not absolutely correct so that you can build a position of trust”* (Supplier 1, medium company).

 Retailers felt it important to have this objective stance. They described it as dangerous to take just one view, and that talking to other suppliers is important. Integrity is a key feature of the relationship, although it is notable that suppliers described it to be part of a longer-term process. Taking short term risks such as disagreeing with a retailer can lead to gains in the future:

 “*I think that the best way to build a relationship is to basically to have the integrity to work with the customer to behave with honesty and so develop relationships based on trust. It could be that integrity leads to stand offs because, and it has done [in] the past as we are not going to promote the products or even reduce the price as we don’t believe it’s right at the moment for the category. So, by applying higher degrees of integrity by refusing to promote or price reducing you may lose a bit of business but if we don’t believe it’s right then it is the right thing to do for the category. In the longer term you will gain sales and category growth”* (Supplier 1, medium company).

 We also found some evidence of concerns about the regulatory frameworks governing the grocery market as factors that constrained category management, although the exact nature of these was unclear:

 “*Sometimes if a retailer has an ad-hoc problem they want to fix then multiple suppliers will work on that. I understand the logic of working together, but from a legal perspective we cannot work together. Unless we are forced to go, I find these together sessions not very useful, as no one talks or inputs not to break the law or say anything of interest to their competitors”* (supplier 6, large company).

 “*I would love the category manager to be in-house so spend some time with the retailers to gain their trust and develop a commercial understanding from the retailers. I know it is dodgy from a legal perspective but if the CM does not understand the basics such as profit condition, how can I help them by unlocking a plan if it does not tick every box, for example Retail Sales Value” *(Supplier 6, large company).

 To mitigate the commercial risks of category management, some supermarkets take action to prevent retailer-supplier relationships becoming too close by moving buyers around:

 “*You know we talk to some [supermarket name] buyers who look after 20 categories and move around fast and it’s always been the case that they will never keep a buyer there for more than 18 months as they don’t want them to build a relationship with a supplier and it becomes cosy, cosy”* (Supplier 2, small company).

###  Theme 4: Competitive Advantages of Category Captaincy and Consequences For Food Purchasing, Diet and Health

 Nodes in theme 4 are coloured green in the Figure. A range of responses were given by interviewees when asked about the role of the “category captain” and the implications of this role. Some suggested that it no longer exists for sound commercial reasons:

 “*It used to exist formally […] up to a point where the Competitive [sic] Commission said that it is not fair on the other suppliers; and particularly in the good old days they would pay for captaincy. Those days have gone, and I think that the market is moving further and further away from retailers selecting only one partner. This is because if you are quite savvy, if you are a buyer and you have four people giving me advice why would I just choose one supplier’s advice. Gone are the days of probably being the only person that is around the table giving advice; most big businesses would accept that. I think the point of difference is that if you have got better advice of more in-depth consumer or shopper knowledge that your competitors perhaps haven’t then they are more likely to listen to you”* (Supplier 8, large company).

 Others indicated that the role still exist, although may now be described using different terms such as “category leader” or “preferred supplier”:

 “*When we talk about collaboration, the word captain is a bit of a dirty word often […] (when asked about the evolution of the role) the role does exist, and the words are being used within the sector”* (Retailer 5, large company)

 “*I think you need to be the category leader, […] to create inspiration and […] to have forward thinkingness more than your competitors”* (Retailer 5, large company).

 The functions of the role were clearly articulated in many accounts, exemplifying the benefits for suppliers, such as greater access to the retailer, greater influence over the category and preferred access to premium shelf space:

 “*I think the category captain will always disproportionally win mainly because of the time spent with ‘time poor’ buyers and retailers and maintain the most contact time”* (Supplier 1, medium company).

 “*So while it should be based on fairly factual information, and I am not saying it’s not, it can be slanted towards the desired outcome towards the wishes of the category captain. I am a bit cynical but that’s how it is”* (Supplier 3, small company).

 “*I know we were in a different situation here, but the big players are paying big sums of money to locate their products at eye level and in the right locations. Basically, blocking our space and then the buyer would get significant margin gains with products in key locations. That is how it works, but the category captain is a label where they are given a seat at the table, and sometimes more access to the data than the others”* (Supplier 1, medium company).

 This view was contradicted by some, including this small supplier:

 “*It might be a buyer has always worked with one of them and will always go with that one for questions and things like that. I don’t think it necessarily hinders us as a small supplier, […] but it does not make any difference to our relationship and involvement with the retailer. We are always working to earn our seat at the table, and being the captain does not open more doors”* (Supplier 9, small company).

 However, there was a feeling among some that, despite a lack of perceptible preferential treatment by retailers, the fact remained that a category captain spent more time with the retailer, which was advantageous:

 “*We do feel they [the retailer] are however more open with the category captain, but that’s just a gut feel rather than on evidence or fact. I sit in reception say at [supermarket name] head office and listen to [Brand X’s] discussions and it does not seem they are treated any differently. They have more ongoing dialogue, so we will put a lot into that meeting whereas category captains probably do not as they will be back in front of the buyer next week rather than like for us just twice a year”* (Supplier 5, small company).

 We found many references to how the role of the category captain was awarded to larger suppliers, with greater access to resources, which could be spent on data, staff and other benefits for retailers, and which were used to the supplier’s commercial advantage (See theme 2):

 “*The secret to being a good category captain is you have got to have the data, the people and the systems to manipulate it”* (retailer 4, large company).

 “*Often you will find that the captain has access to EPOS [supermarket electronic point of sale] data at different levels to the rest of the category management team including other suppliers so cynically can use that data for competitive advantage and read into it whatever they want. This enables them to throw out any results which look best for them. At the end of the day, they are investing much more into the relationship as they have a much bigger team and resource invested into this retailer and they have to make it work. That has to be paid for, and the only way this can be paid for is by them having the biggest turnover and profit from the wider category teams”* (Supplier 1, medium company).

 “*I have heard [that the] category captain will weave the data to make it say something to put other suppliers off the scent. To protect their business they have to ‘weave’ the data as best they can which takes even more time to do” *(Supplier 1, medium company).

 The interviews revealed some details of how category captains were appointed. Accounts pointed towards the privileged position of the largest suppliers, and the payment of resources (financial or in kind) for the role, as well as the advantages it brought in terms of influence:

 “*Generally, the category captaincy does come with a commercial contribution. We bought our way in, the situation changed and the year after we still maintained the same level of interaction with the buyer. The relationship had been built. The buyer trusted the individuals and the relationship with [supplier name] […] liked the relationship and therefore kept the contact as before” *(Supplier 4, large company).

 “*With commercial discussions there is a payment to be made; this can be either direct or indirect. There are then more costs associated with being a category captain I would say. Clearly if you are doing it properly you would need to put more resource into it from a people perspective. It might not be more heads, but if you suddenly become the category captain; if you were only seeing them a couple of times a year this might now become monthly or even weekly. Suddenly you need more heads or horsepower. You may need to buy more data, so to be a true partner and they need extra data you will have to purchase this regardless of cost. And you may feel it’s more appropriate to get closer to that retailer and even get ‘under the retailer’s skin.’ So, it comes with cost”* (Supplier 4, large company).

 The risks of the role were also highlighted by one retailer:

 “*You can tell if you go in a retailer if they have been ‘bought’ by a supplier, where the index for that supplier is great, they have a lot of space and probably getting profit on the back of that from the ‘back margin’ rather than the ‘front margin.’ So that is a definite watch out, you know you have gone too far when you start seeing stuff that is out of proportion”* (Retailer 4, large company).

 Finally, the preferred access to premium shelf space gained by category captains and the bigger brands (suppliers) was recognised by some interviewees to lead to distortion of food purchasing through preferential placement and promotions, with potential consequences for health:

 “*A shift also back to more branded products due to the strength of branded promotions as they are fighting very hard to get back their share of the market. They have lost share over the last 10 years and fighting very hard to get that back […] that entices people as promotions are promotions. They are big and powerful on a gondola end, they look great, and people will pick the products up, […] products have to be in the right place in the store for the consumer to see it. […] So that’s how private label quite often build its sales, when you go in the store there will always be massive displays of the brands and own label tucked in the corer out of the way”* (Supplier 3, small company).

 “*I think the biggest challenge to category management is the way traditionally it is being used. I am cynical about the amount of honesty that exists within the relationship, and I think because it is such a commercial relationship. For years we have seen as part of category management and the need to grow and develop a category i.e. a promotional strategy, say buy one get one free which is not always necessary for the consumer. Not every consumer wants a free product, they would probably be happier with a percentage saving of the base product. These kinds of deals on say crisps can have health implications as they encourage obesity for young people”* (Supplier 1, medium company).

## Discussion

###  Summary of Main Findings

 This is the first paper, to our knowledge, to assess, from a public health perspective, how category management—a central driver of the UK grocery system—works in practice, how it tends to favour the largest suppliers, and how this may influence food purchasing, diets and health. The findings of this study, together with research on food availability in supermarkets, suggests that these commercial behaviours sustain a mix of food products available to consumers that maximise sales and profits, but are poorly aligned with the interests of population health. These practices appear to be historically rooted, longstanding and ingrained. The findings support claims that the GSCOP has helped to reduce the undue influence of the biggest food manufacturers on food retailing, turning category management into a collaborative supplier-retailer venture aimed at mutual value creation. However, because the GSCOP is only concerned with commercial competition, it fails to address the huge external costs that the food system imposes on health, which also represent a burden on consumers.

 In secondary analysis of 19 interviews with key stakeholders from both major suppliers (manufacturers) and retailers (supermarkets), four substantive themes were found. The first theme focussed on the characteristics of a successful category management relationship between retailer and supplier, identifying factors that build trust and rapport between the parties as of central importance. The second theme focussed on the virtuous circle of category management, including the ways in which suppliers invest in retailers to create shared value. It highlights how such investment leads to indebtedness and reciprocity, creating reinforcing feedback loops that sustain the system. Counterbalancing this are negative feedback pathways characterised by the constraining influences of regulation and commercial risk on category management. The fourth theme focuses on the competitive advantages of category captaincy and consequences for food purchasing, diet and health.

###  Strengths and Limitations

 A strength of this paper is the interdisciplinary approach we have adopted, bringing together perspectives and expertise from public health, food policy, psychology and business studies to provide unique insights into the workings of UK supermarkets, and impacts on public health. Interviewees represented retailers and suppliers from small, medium and large companies, including grocery retailers that are subject to the GSCOP. This allowed us to solicit information from individuals with diverse experience in the field, and who had direct experience within category management. MB adopted a phenomenological approach throughout the interviews, including the requirement to disregard previous knowledge and understanding, so as to avoid bias; however, they accepted that this was difficult at times.

 As with all interview studies, we were reliant on participants providing honest and accurate accounts from their own perspective. However, such studies are subject to biases that influences responses, including social desirability, recall and sponsor bias. The latter, in particular, may have been at play, with participants feeling that they needed to respond in ways that conformed with their employer’s policy rather than their own views. That said, many interviewees spoke candidly about their experiences and none mentioned company policy.

 A limitation of this study is the use of secondary analysis. The data was analysed for a purpose beyond the original research question and therefore may not yield data that is relevant. However, this was not the case in this study. Secondary analysis can also lose some inference of the “unsaid”, pauses, body language etc. This was mitigated as much as possible as the research team included MB, who conducted the original interviews, and the team worked closely on the analysis. The interviews were conducted in 2016-2018. The study should therefore be viewed within this context and not all insights may be of contemporary relevance.

 When conducting analysis, differences in size of company were not taken directly into consideration, and doing so may have allowed for a different perspective. However, the aim of this paper was not to compare views across company size, it was to assess views of individuals from a range of companies, and during analysis, the salience of perspectives was analysed. Nevertheless, interviewees made many comments about smaller and larger suppliers, in particular with reference to the differential influence of such scale on category management, and these views were important in our analysis.

###  What the Study Adds to Prior Knowledge

 We set out to understand the nature of the relationship between retailers and their suppliers and the implications of this relationship for the overall healthiness of the food offer in supermarkets. We generated detailed knowledge on the category management relationship (themes 1-4), but less information concerning the implications for the healthiness of the food offer (theme 4). This is perhaps unsurprising, as the interviews were primarily conducted to explore value creation in category management relationships and had no specific focus on diet or health.

 We revealed the complex web of factors that contribute to successful category management relationships between suppliers and retailers, as well as expectations and obligations of both parties that lead to indebtedness and reciprocity, described by one retailer as a “virtuous circle.” This co-dependent relationship that underpins category management ultimately results in differential power among the parties. The retailer’s “buyer” was described as ultimately being in power and will choose which suppliers to give their time to. Suppliers described being able to “buy in” to the retailer’s world by becoming a “category captain” with a direct or indirect commercial investment, including staff, data, marketing etc. While the term category captain was not used by all interviewees, some preferring newer terms such as “trusted partner,” “category leader” or “preferred supplier,” it remained very prominent in the discourse of participants, suggesting that it is a term still widely used that carries currency in the category management world.

 Retailers were described as being time-poor, and responsible for many categories, so will therefore lean on suppliers’ category managers and captains provide market insights to help them make decisions. Thus, the supplier and retailer become locked together in a mutually beneficial relationship; the retailer does not want to lose the supplier’s investment in infrastructure, supportive personnel (“implants”) and data, and the supplier wants to remain the category captain or “trusted supplier” because of the significant competitive advantage offers.

 The supplier-retailer relationship is thus highly transactional and, whilst being described as a “triple win,” evidence from these interviews suggests that sales and profits are the key priority for retailers and suppliers. Suppliers go to great lengths to achieve the role of preferred supplier, and will offer retailers exclusivity deals and stock, while other suppliers struggle to gain access. Retailers describe knowing that they are being “looked after” by preferred suppliers. For example, category captains give more access to data (which is often presented in a way that sheds favourable light on their brand – with acknowledged bias) and more time with the retailer, so they are able to use both internal and external insights to secure the best shelf space.

 There are clear reasons why suppliers are willing to invest so much into this relationship.^15^ Shelf space is monopolised by the biggest brands, with suggestions in our data that bigger suppliers have been able to invest significantly to ensure their products are located at eye level and in key locations. Interviewees suggested that whoever has the “best story and the best motivation” will get the space in prime locations. Following legislation in 2022 that led to removal of HFSS products from the ends of aisles and checkouts,^25^ this prime space *within* aisles (eg, at eye level) has become even more desirable, and we have seen a range of novel tactics emerge to gain product prominence (eg, theatrical, framed displays of branded confectionery), as well as significant promotions (eg, mid-aisle pallet stacks of branded confectionery). While these tactics do not necessarily flout the new regulations, they are clearly against the spirit and intent of them (ie, to reduce pressure on consumer to buy unhealthy foods).^26^

 The 2009 Competition Commission report^11^ acknowledged the role of grocery retailers in public health, with the Food Poverty Project highlighting that reduced choice in supermarkets was limiting customers’ choice of healthy food options, particularly for those from more disadvantaged backgrounds. It also suggested that contributing factors come from a dominance of processed food particularly in larger grocery retailers. The Competition Commission admitted that their scope of work was limited to competition matters only and matters such as public health concerns should be assessed by relevant government departments (eg, Department of Health and Social Care [DHSC], Department for Environment, Food & Rural Affairs [Defra] or The Food Standards Agency [FSA]). Our findings suggest that the current GSCOP (last reviewed in 2009) seems likely in part to have achieved its main goal: to ensure that large grocery retailers treat their direct suppliers fairly and lawfully. Yet, we also found that there remained evidence of payments between suppliers and retailers for marketing, volume sales, wastage and other aspects of listing their products, which the GSCOP aimed to eradicate. Such costs were only affordable by the largest suppliers. This suggests that the GSCOP has not entirely created the level playing field it intended. Previous research suggest that the use of category captains improves sales, but competitors often suffer.^13^ Our results were consistent with these findings.

 Jack’s detailed report on the supermarket business model identified the challenging economics of the market, which has become highly dependent on so-called commercial income – the payments made by suppliers to list their products on shelves.^27^ With suppliers increasingly competing for (premium) shelf space, especially since the 2022 Promotion and Placement Regulations,^25^ retailers have become more powerful, with greater bargaining power over suppliers. In such conditions, it is unsurprising that the biggest brands are able to command greater influence in category management, secure more profitable shelf space and higher sales.

 Importantly, the GSCOP remains entirely focussed on commercial competition and does not address the wider (societal) problem that category management generates – that the advantages open to the largest suppliers lead to preferential placement and sales of the least healthy, processed foods, and these have not been addressed by the 2022 Promotion and Placement Regulation from DHSC.^25^

###  Interpretation and Implications for Policy-Makers

 Evidence is building that the public is not in support of marketing strategies that promote unhealthy foods, and such tactics are not what consumers want.^28^ The data in this paper mention value for the customer, and the “triple win” of value creation for supplier, retailer and consumer. However, retailer marketing strategies are widely seen as problematic for consumers, especially parents; they notice that supermarkets are designed in ways that manipulate buyers and promote unhealthy options in ways that cannot be avoided (eg, through excessive numbers of facings of unhealthy products, often at eye level).^29^ The main aim of the CMA is to promote competition and protect consumers for the benefit of the UK economy and its people. While this is inherent in the spirit of the GSCOP, in practice the existing regulatory framework lets down the public by failing to take account of the social (including health) impacts of the ways supermarkets operate with regard to category management.

 An argument regularly made by industry is that consumers need more education and should have the opportunity to make their own choices. However, the present arrangements in supermarkets restrict choices, especially affordable, healthy choices.^30^ We live in an obesogenic environment, where unhealthy food is more often and more heavily promoted,^31^ and individuals are marketed unhealthy products that they do not necessarily need or want, but are relatively cheap. Defra is presently developing a new cross-government food strategy for the UK. A key driver of unhealthy food sales is category management, which determines the product mix and display in supermarkets. Government needs to take action to ensure that health considerations are taken into account in regulating these practices.

###  Unanswered Questions and Further Research

 It would be informative to engage industry in further discussion of category captaincy and its potential leverage in creating a supermarket environment that is more conducive to better public health. Similar work should be conducted with respect to achieving sustainability goals, with which there are important overlaps with population health.^32^ Research is also needed to explore the views of policy-makers in government and the CMA concerning the disadvantageous nature of the current regulatory framework for public health. There have been many calls for UK food strategy to become less siloed and more joined up. Category management is another, as yet unrecognised facet of this problem, in which health and commercial policy-making needs to become more closely aligned.

 Conducting further interviews in the current landscape would also be beneficial in order to assess the changes that may have taken place, especially during a cost-of-living crisis and since the implementation of the government’s Promotion and Placement Regulations of HFSS foods in October 2022.^25^

## Conclusions

 Unhealthy diets are highly prevalent and result in a huge burden on the NHS and society. Around 80% of the food eaten in the UK comes from supermarkets, which are therefore the most important food retail environment that could be leveraged to improve population diets and health. However, maximising sales and profits remains at the heart of all retailing decisions in a highly competitive market. As it stands, there is no reason for one individual supermarket to change, as they would lose their competitive advantage. Ultimately, supermarkets and major brands are playing a game with society that they will continue to win at the expense of the population and planetary health until either the prevailing economic paradigm changes,^33^ or current regulation of the supplier-retailer relationship is changed. Significantly closer scrutiny of this relationship is needed across government by the CMA, DHSC and Defra. The GSCOP is intended to ensure that retailers are acting in a way that allows for fair business competition. Our data suggest that the current legislation is not fit for purpose. We have provided evidence that the nature of category management leads to the disproportionate promotion and sales of unhealthy foods by the biggest brands, which is not in the interests of consumers or population health.

## Acknowledgements

 We would like to thank all interviewees who took part in the initial study. We are grateful to Chris Holmes (independent consultant and former food industry executive), Emma Coles (independent consultant and former food industry executive), Amandine Garde (Professor of Public Health Law, University of Liverpool) Sujitha Subramanian (Reader in Law, University of Liverpool), Leigh Sparks (Professor of Marketing, University of Stirling), and Jaideep Prabhu(Professor of Marketing, University of Cambridge).

## Disclosure of artificial intelligence (AI) use

 Not applicable.

## Ethical issues

 The original study was approved by Sheffield Hallam University (SHU) ethics committee, and further approval was sought for secondary analysis (REF/ER69211078).

## Conflicts of interest

 Martin White received honoraria for: delivering the Food for Health programme for government officials from low and middle income countries, in association with the Judge Business School, University of Cambridge, UK, funded by Bloomberg Philanthropies; delivering a workshop and associated work to inform a food policy strategy for the States of Jersey, 2023-2024; honoraria for delivering a workshop and associated work to inform a food policy strategy for the Guernsey Health Improvement Commission during 2024; and honoraria for acting as a specialist adviser to the House of Lords Committee on Food Diet and Obesity in 2024. Martin White is also an unpaid expert adviser to the Food Foundation, a non-governmental organisation that advocates for healthier food retail environments. None of the above contributed to the design or funding of the current manuscript. Roxanne Armstrong-Moore and Michael Benson declare no relationships or other interests.
